# Gp78 deficiency in hepatocytes alleviates hepatic ischemia-reperfusion injury via suppressing ACSL4-mediated ferroptosis

**DOI:** 10.1038/s41419-023-06294-x

**Published:** 2023-12-08

**Authors:** Changbiao Li, Yichao Wu, Kangchen Chen, Ronggao Chen, Shengjun Xu, Beng Yang, Zhengxing Lian, Xiaodong Wang, Kai Wang, Haiyang Xie, Shusen Zheng, Zhikun Liu, Di Wang, Xiao Xu

**Affiliations:** 1 Zhejiang University School of Medicine, Hangzhou, 310058 China; 2 NHC Key Laboratory of Combined Multi-organ Transplantation, Hangzhou, 310003 China; 3 Key Laboratory of Integrated Oncology and Intelligent Medicine of Zhejiang Province, Hangzhou, 310006 China; 4 Department of Hepatobiliary and Pancreatic Surgery, First Affiliated Hospital of Zhejiang University School of Medicine, Hangzhou, 310003 China; 5 The Fourth School of Clinical Medicine, Zhejiang Chinese Medical University, Hangzhou, 310053 China; 6 Department of Hepatobiliary and Pancreatic Surgery, Shulan (Hangzhou) Hospital, Hangzhou, 311112 China; 7 Institute of Immunology and Sir Run Run Shaw Hospital, Zhejiang University School of Medicine, Hangzhou, 310058 China; 8 Liangzhu Laboratory, Zhejiang University Medical Center, 1369 West Wenyi Road, Hangzhou, China

**Keywords:** Pathogenesis, Cell biology

## Abstract

Ferroptosis, which is driven by iron-dependent lipid peroxidation, plays an essential role in liver ischemia-reperfusion injury (IRI) during liver transplantation (LT). Gp78, an E3 ligase, has been implicated in lipid metabolism and inflammation. However, its role in liver IRI and ferroptosis remains unknown. Here, hepatocyte-specific gp78 knockout (HKO) or overexpressed (OE) mice were generated to examine the effect of gp78 on liver IRI, and a multi-omics approach (transcriptomics, proteomics, and metabolomics) was performed to explore the potential mechanism. Gp78 expression decreased after reperfusion in LT patients and mice with IRI, and gp78 expression was positively correlated with liver damage. Gp78 absence from hepatocytes alleviated liver damage in mice with IRI, ameliorating inflammation. However, mice with hepatic gp78 overexpression showed the opposite phenotype. Mechanistically, gp78 overexpression disturbed lipid homeostasis, remodeling polyunsaturated fatty acid (PUFA) metabolism, causing oxidized lipids accumulation and ferroptosis, partly by promoting ACSL4 expression. Chemical inhibition of ferroptosis or ACSL4 abrogated the effects of gp78 on ferroptosis and liver IRI. Our findings reveal a role of gp78 in liver IRI pathogenesis and uncover a mechanism by which gp78 promotes hepatocyte ferroptosis by ACSL4, suggesting the gp78-ACSL4 axis as a feasible target for the treatment of IRI-associated liver damage.

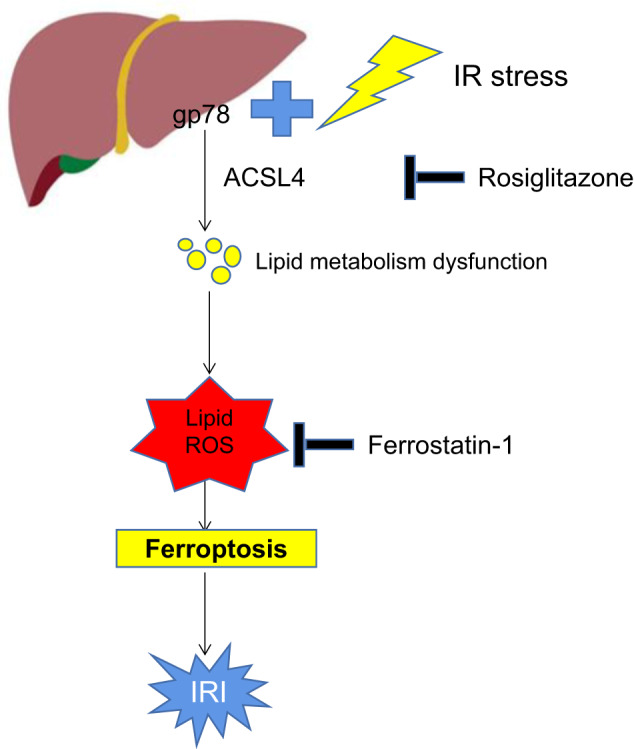

## Introduction

Liver transplantation (LT) is an established life-saving treatment for end-stage liver diseases and early hepatocellular carcinoma (HCC) [1–5]. During LT, the liver is subjected to ischemia and reperfusion injury (IRI), leading to early allograft dysfunction (EAD) and even primary graft nonfunction (PNF), compromising the survival of grafts and recipients, especially for extended criteria donors [6–8]. Despite great clinical significance, there is no effective treatment for liver IRI yet, and about 25% of LT recipients still develop EAD. Thus, it’s of great significance to understand the pathogenesis of liver IRI to improve the outcomes of LT recipients [9, 10].

IRI was considered to be caused by metabolic stress during ischemia stages and oxidative burst after reperfusion inducing severe cell death and sterile inflammation. Multiple inflammatory cell death has been reported to contribute to liver injury, including pyroptosis, necroptosis, autophagy, as well as ferroptosis [11–15].

Ferroptosis is a novel programmed cell death that is iron-dependent and driven by selective lipid peroxidation [16]. As a pathological cell death, ferroptosis has been implicated in liver IRI [17]. Iron overload was a risk factor for hepatic IRI in LT, and iron chelator or lipid peroxide scavenger alleviated liver damage in a mouse hepatic I/R model [11, 18, 19]. Therefore, targeting ferroptosis might be a potential therapeutic strategy for liver IRI. However, the underlying mechanisms of ferroptosis in liver IRI are not fully understood [20]. Emerging evidence indicates that lipid metabolism is fundamental for shaping ferroptosis response, remodeling lipid composition might alleviate ferroptosis and liver IRI [21].

Gp78 (also known as AMFR) was initially described as a cell surface receptor for the autocrine motility factor (AMF), a tumor-secreted cytokine. It also exhibits E3 ligase activity at the ER membrane, mediating polyubiquitination of diverse substrates, including the cholesterol metabolism regulatory proteins HMGCR and INSIG-1, altering lipid metabolism [22–24]. Further, gp78 is highly expressed in mouse hepatocytes, and gp78 overexpression promotes hepatic lipid accumulation, inflammation, and subsequent development of nonalcoholic fatty liver disease (NAFLD) [24]. Apart from lipid metabolism, gp78 is involved in many important physiological and pathological functions, including cell apoptosis, inflammation and oxidative stress [25–27]. These findings suggest that hepatocyte gp78 may play a critical role in regulating liver function. However, the involvement of gp78 in hepatic I/R injury remains poorly understood.

In this study, our findings first demonstrated that the expression of gp78 in donor livers was positively correlated with hepatic damage after LT. Further, we investigated the functional role of gp78 in liver IRI using hepatocyte-specific gp78 knockout or overexpressed mice. In mice, hepatocytic gp78 overexpression aggravated liver IRI while hepatocyte-specific knockout of gp78 attenuated liver IRI. The multi-omics analysis (transcriptomics, proteomics, and metabolomics) was conducted to explore the potential mechanisms. And we unveiled the supportive role of gp78 in ferroptosis, and this effect was partly mediated by remodeling lipid metabolism through ACSL4. These findings contribute to our understanding of the mechanism of ferroptosis in liver IRI, and the gp78-ACSL4 axis may be a potential therapeutic target.

## Materials and methods

### Bioinformatic analysis of IRI-related genes in GSE15480

The Gene Expression Omnibus (GEO) database is a public gene expression database created by the National Center for Biotechnology Information (NCBI). We selected the dataset GSE15480 which includes 12 LT recipients, and performed the differential analysis of gene expression data in GSE15480 dataset by the GEO2R online tool in the GEO database. Only those genes with adjusted *P*-value < 0.05 and |log_2_fold change| ≥ 0.5 were considered differentially expressed genes (DEGs). The Gene Ontology (GO) and Kyoto Encyclopedia of Genes and Genomes (KEGG) enrichment of DEGs were conducted on David website (https://david.ncifcrf.gov), and the Sangerbox website (www.sangerbox.com) was used to draw volcano plots and bubble plots of GO and KEGG enrichment for data visualization.

### Patients and liver biopsies

Human liver samples were collected at the First Affiliated Hospital of Zhejiang University School of Medicine and Shulan Hospital (Hangzhou, China) from March 10th to October 30th 2022. According to standard operating procedures, the donor liver was evaluated, trimmed, an orthotopic LT was performed. Donor livers were stored in UW solution prior to LT. The pretransplant liver specimens were obtained from the left lobe of the liver when trimming the donor liver. The posttransplant liver specimens were acquired prior to the abdominal closure. To evaluate liver function, recipients’ serum alanine aminotransferase (ALT) and aspartate aminotransferase (AST) after LT within 72 h were obtained from the inpatient register system. The etiology of patients undergoing LT included advanced liver cirrhosis and hepatocellular carcinoma, and the information of LT patients were summarized in Table S1. Liver samples were subject to immunohistochemistry (IHC) described below in detail. The IHC scores of gp78 was based on positive strength and areas, and the positive strength and areas increased from 1 to 4. 1 and 2 were considered as low expression, 3 and 4 were considered as high expression. Every liver biopsy was scored by different pathologists independently. The IHC scores of every liver biopsy were summarized in Fig. S1. This study has been approved by the First Affiliated Hospital of Zhejiang University School of Medicine. The participants or their guardians provided signed consent.

### Mice

Male C57BL/6 mice (8–10 weeks old) were purchased from Hangzhou Medical College (Hangzhou, China). Gp78^flox/flox^ mice were kindly provided by Professor Baoliang Song. Gp78^flox/flox^ mice were crossed with Albumin-cre mice (gempharmatec, China) to generate hepatocyte-specific gp78 knockout mice (gp78 HKO), whose genotypes were Alb cre^+^ gp78^flox/flox^. PCR-based genotyping was performed using the primer pairs as shown in Table S2. All mice in this study were bred in a standard environment with 12 h light/dark cycles. All procedures related to animals were reviewed and approved by the Institutional Animal Care and Use Committee of the First Affiliated Hospital of Zhejiang University School of Medicine (Reference Number: 2019678).

### Chemicals

Ferrostatin-1 and Rosiglitazone were produced by MedChemExpress (Newark, NJ, USA). For mice treatments, mice were randomly divided into control treatment and drug treatment group. Rosiglitazone and Ferrostatin-1 are dissolved in DMSO, diluted by PEG300, Tween 80 and normal saline, then intravenous injected at 5 mg/kg and intraperitoneally injected at 10 mg/kg 1 h before IR treatments, respectively.

### Adeno-associated virus (AAV)

For overexpressing gp78 in vivo, the animals were randomly divided into two groups: overexpression group and control group for injecting AAV. The mice of overexpression group were injected in the lateral tail vein with a dose of 1.3 × 10^11^ genome copy (GC) of AAV8-TBG-gp78 (WZ Bioscience Inc, China). And a dose of 1.3 × 10^11^ GC of AAV8- GFP was injected into the lateral tail vein of the control groups. After 4 weeks of recovery, I/R surgery was conducted.

### Hepatic I/R mice model and treatment

In brief, the sham group only had free hepatic blood vessels after laparotomy, and did not block blood flow. The hepatic I/R group was freed from the hepatic vessels and the blood supply to the left lobe and mid-hepatic lobe was blocked for 90 min. Then the blood vessels were opened for 6 h. The numbers of mice vary from 4 to 8 in each group, if the mice died during operation or resuscitation, the samples will be excluded. All the operations were performed by the same operator.

### Liver function analysis

Serum levels of ALT and AST were determined using commercial kits (elabscience, China). All kits were used according to manufacturer instructions.

### Hematoxylin and eosin (H&E) staining

Liver samples were fixed in 4% paraformaldehyde, dehydrated, and embedded in paraffin. Paraffin-embedded liver Section (4 μm) were deparaffinized with xylene, rehydrated using an ethanol gradient, and stained with H&E. Images were acquired using a light microscope (Olympus, Japan).

### TUNEL staining

TUNEL staining (Servicebio, China) was performed to evaluate cell death in liver tissue slides, according to manufacturer instructions. The slides were viewed under a laser scanning confocal microscope (LSM800; Zeiss, Germany).

### IHC staining

Liver samples were fixed in 4% paraformaldehyde, dehydrated, and embedded in paraffin. The liver sections were treated with 3% H_2_O_2_ for 10 min and then blocked with 10% BSA for 1 h at 37 °C. The sections were incubated with primary antibody (Servicebio, China) overnight at 4 °C, followed by incubation with biotinylated secondary antibodies (Cat No. AP180B; 1:200, Millipore, USA) for 1 h at 37 °C. Subsequently, the sections were incubated with horseradish peroxidase (HRP)–labeled avidin (Cat No. SA-5004; 1:100, Vector Laboratories, USA) for 30 min. Finally, the sections were visualized by staining with 3,3-diaminobenzidine (Zhongshan Golden Bridge Biological Technology Co., Ltd., China) and counterstained with hematoxylin. The sections were viewed under a light microscope (Olympus, Japan).The antibodies used in IHC are listed in Table S3.

### Detection of iron and malondialdehyde (MDA)

The iron concentration was assessed using the Iron Assay Kit (Beyotime Biotechnology). The hepatic MDA concentration was measured using the MDA Assay Kit (Sorabio Biotechnology, China). All kits were used according to manufacturer instructions.

### Quantitative real-time PCR and western blot analysis

Total RNA was isolated from liver tissues using Nucleozol Reagent (MNG, Germany) according to manufacturer instructions. RNA (1 μg) was reverse transcribed into cDNA using the First Strand cDNA Synthesis Kit (Vazyme, China) and real-time PCR was performed using the SYBR Green PCR Master Mix (Vazyme, China) on an ABI 7500 fast Real-time PCR Detection System (Bio-Rad Laboratories, USA). The mRNA expression was normalized to that of β-actin. The primer sequences used are listed in Supporting Table S2.

Western blotting was performed as previously described. Briefly, total proteins were extracted from tissues using radioimmunoprecipitation assay lysis buffer containing PMSF, protease inhibitor cocktail and phosphatase inhibitor (Fudebio, China). The protein concentration was quantified using the BCA Protein Assay Kit (Thermo Fisher Scientific, USA). The samples were centrifuged at 12,000 × *g* for 10 min, resuspended in SDS loading buffer, and boiled at 95 °C for 10 min. Proteins were separated by SDS-PAGE and transferred to polyvinylidene fluoride membranes (Millipore, USA). The membranes were blocked with 5% nonfat milk and incubated with primary antibodies overnight at 4 °C. Subsequently, the membranes were incubated with HRP-conjugated secondary antibodies for 1 h at room temperature and developed with ECL reagent (Fudebio, China). The images were captured by the ChemiDoc MP Imaging System (Bio-Rad, USA). β-actin served as the internal control. The antibodies used in western blotting are listed in Table S3.

### Cytokines and chemokines assays

Inflammatory cytokines (TNF-α, IL-1β) in serum were measured by commercially available enzyme-linked immunosorbent assay kits (Proteintech, China). All kits were used according to manufacturer instructions.

### Metabolomic, proteomic and transcriptomic analysis

Liver samples were obtained from the gp78-overexpressed mice and controlled WT mice liver tissues subjected to IR injury. Sample preparation, extraction and detection for transcriptomic, proteomic and metabolomic sequencing was performed by Metware Biotechnology Co., Ltd. (Wuhan, China) as previously reported [28]. Obtain and visualize differentially expressed genes as *p* values less than 0.05 and a fold change greater than 1.5 (for transcriptomic analysis) or 1.2 (for proteomic analysis). And significant dysregulated metabolites were screened by VIP > 1 and *p* value < 0.05.

### Immunofluorescence (IF) staining

Liver samples were fixed in 4% paraformaldehyde, dehydrated, and embedded in paraffin. Immunofluorescence staining was performed to detect targets using antibodies: anti-gp78 (16675-1-AP, Proteintech, China), anti-HNF4α (Ab41898, Abcam, UK) at 4 °C overnight. After being incubated with fluorophore-conjugated secondary antibody (A-11034, A-21424, Invitrogen, USA), slices were counterstained with DAPI (Ab104139, Invitrogen, USA). Finally, the fluorescence staining results were observed by confocal microscope (LSM710, Carl Zeiss Microscopy, Germany).

### Statistical analyses

All assays were conducted at least 3 times and reproducible results were obtained. If the data conform to normal distribution, the results are presented as mean ± Standard Deviation (SD), and the statistical significance between different groups was analyzed by paired Student’s t test or unpaired two-tailed Student’s t test. If not, the results are presented as median ± interquartile, the statistical significance was analyzed by nonparametric test (Prism; GraphPad). Statistical significance was defined as *p < 0.05; **p < 0.01.

## Results

### Gp78 expression in donor livers correlates with the severity of hepatic damage after LT

**Fig. 1:**
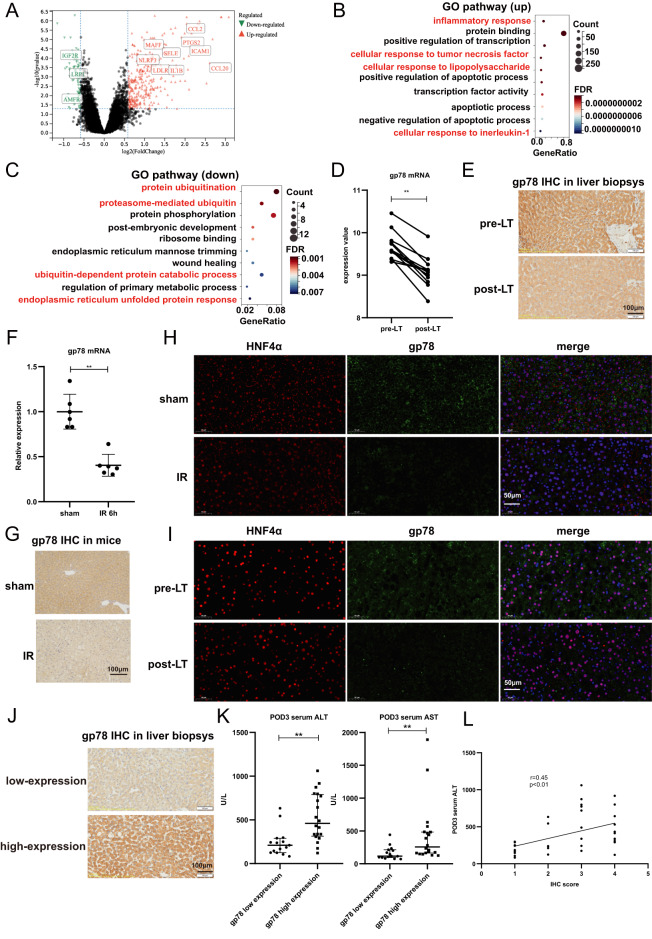
Gp78 expression is decreased in hepatic IRI and positively correlates with hepatic damage in liver transplantation recipients. **A** The volcano map of mRNA expressions from GEO datasets (GSE15480). **B** The bubble graph of GO enrichment of upregulated DEGs in GSE15480. **C** The bubble graph of GO enrichment of downregulated DEGs in GSE15480. **D** The peri-operative graft expression of gp78 mRNA levels in GSE15480 (*n* = 12/group). **E** Representative IHC staining of gp78 in hepatic biopsies from a human liver transplantation cohort, scale bar = 100 μm. **F** Gp78 mRNA levels in the livers of mice subjected to 90 min of ischemia and subsequent reperfusion for 6 h (*n* = 6/group). **G** Representative IHC staining of gp78 in the livers of mice subjected to 90 min of ischemia and subsequent reperfusion for 6 h, scale bar = 100 μm. **H** Co-staining analysis of HNF4α and gp78 by IF in mice liver samples from sham and liver I/R group, scale bar = 50 μm. **I** Co-staining analysis of HNF4α and gp78 by IF in human liver samples undergoing liver transplantation, scale bar = 50 μm. **J** Representative IHC staining of gp78 low expression and gp78 high expression of hepatic biopsies before liver transplantation, scale bar = 100 μm. **K** The serum ALT and AST levels at POD3 in gp78 low expression (*n* = 15) and gp78 high-expression groups (*n* = 20) of LT recipients. **L** The Pearson’s correlation between IHC scores of pre-LT gp78 expression and serum ALT levels at POD3 (*n* = 35 in total).

We first evaluated global gene expressions in the LT samples of GEO databases (GSE15480) (Fig. 1A). The GO enrichment analysis indicated a significant increase in the expression levels of those genes involved in mediating inflammatory response and a significant decrease in the expression levels of those genes involved in the protein ubiquitination process during LT (Figs. 1B, C). Due to the intimate association of gp78 with the protein ubiquitination, we evaluated the peri-operative graft expression of gp78 in GSE15480, and found that gp78 mRNA expressions were significantly decreased after reperfusion (Fig. 1D). In parallel, we collected 35 pairs of pretransplant (pre-LT) and posttransplant (post-LT) liver biopsies to detect gp78 protein levels by IHC. Similarly, gp78 protein levels were significantly decreased after LT (Fig. 1E). In addition, the hepatic I/R model was successfully established in mice, then gp78 mRNA and protein expression were measured in the liver samples. As shown in Fig. 1F, G, I/R triggered a significant decrease of gp78 in the liver of mice. Since liver tissues consists of many types of cells, including hepatocytes, Kupffer cell, endothelial cells and fibroblasts, we further explored whether gp78 was dysregulated in hepatocytes, the most abundant cells in the liver. First, we explored the expression of gp78 in the “single cell type of livers” of The Human Protein Atlas website (www.proteinatlas.org), it was found that gp78 was mainly expressed in hepatocytes, rather than other cells in the liver. The expression of gp78 in hepatocytes was 2–3 folds of that in Kupffer cells, endothelial cells and fibroblasts (Fig. S2). Next we performed the staining of hepatocyte marker (HNF4α) and gp78 by IF in the mice and human liver samples, and observed that gp78 expression in hepatocytes declined after reperfusion in both mice and human samples (Figs. 1H, I). These observations suggest that hepatic I/R injury leads to the downregulation of gp78 in hepatocytes.

Next, we evaluated the correlation between pre-LT gp78 levels and post-LT hepatic damage (*n* = 35). According to the IHC scores of liver biopsies, we divided the 35 donors into two groups: pre-LT gp78 low-expression group (*n* = 15) and pre-LT gp78 high-expression group (*n* = 20) (Fig. 1J and Fig. S1). Interestingly, serum levels of ALT and AST within post-operation day 3 (POD3) were significantly higher in the pre-LT gp78 high-expression group, suggesting more injury after LT (Fig. 1K), and pre-LT gp78 protein expression in donors was positively correlated with serum ALT levels after LT (Fig. 1L). Together, these data imply gp78 may play a role in the hepatic I/R injury.

### Gp78 deficiency in hepatocytes alleviates liver IRI in mice

**Fig. 2:**
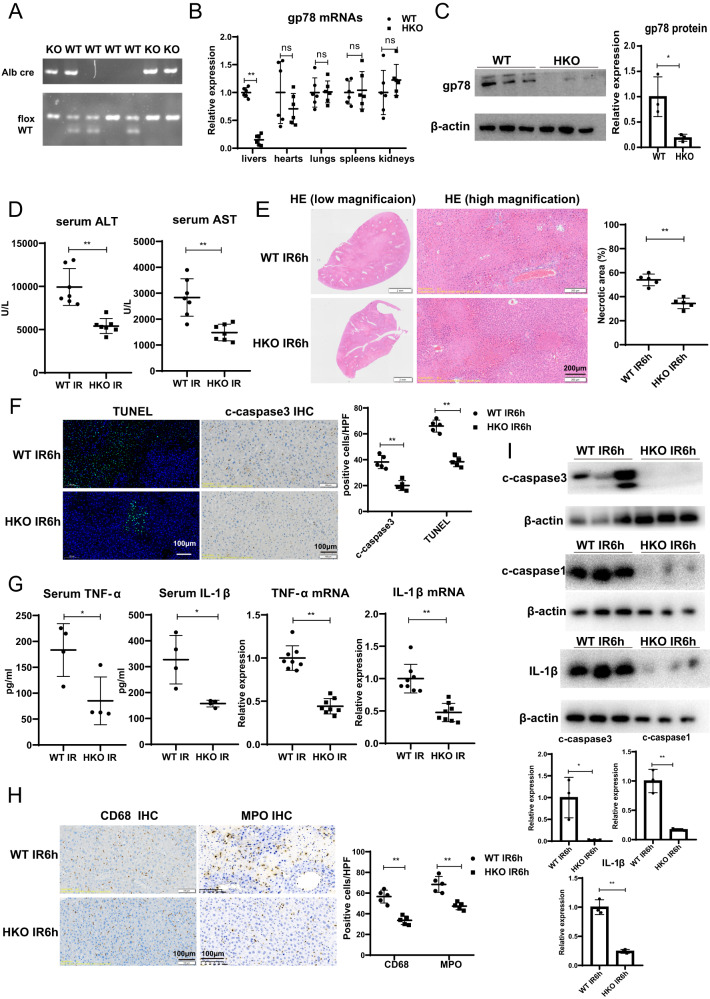
Gp78 deficiency in hepatocytes alleviates liver IRI. **A** The genotypes of Alb cre and flox from wildtype (WT) and hepatocyte-specific gp78 knockout (HKO) mice. **B** Gp78 mRNA levels in different organs including livers, hearts, kidneys, lungs and spleens from WT and gp78 HKO mice (*n* = 6/group). **C** Gp78 protein levels detected by western blot in the livers from WT and gp78 HKO mice (*n* = 3/group). **D** Serum ALT and AST levels in WT and gp78 HKO mice at 6 h after I/R injury (*n* = 7/group). **E** The representative H&E staining images of liver sections from WT and gp78 HKO mice in I/R groups at 6 h post reperfusion (*n* = 5/group), scale bar = 200 μm. **F** The representative images and the statistical quantification of TUNEL staining and IHC staining of cleaved caspase3 in liver sections of WT and gp78 HKO mice at 6 h after I/R (*n* = 5/group), scale bar = 100 μm. **G** The levels of Cytokines (TNF-α and IL-1β) in serum and liver of WT and gp78 HKO mice at 6 h after I/R (*n* = 4/group for serum samples, *n* = 8/group for liver samples). **H** The representative images and the statistical quantification of IHC staining of CD68 and MPO in liver sections of WT and gp78 HKO mice at 6 h after I/R (*n* = 5/group), scale bar = 100 μm. **I** The western blot of cleaved caspase1, cleaved caspase3 and mature IL-1β in WT and gp78 HKO mice at 6 h after I/R (*n* = 3/group).

The positive correlation between gp78 expression and hepatic damage after LT promoted us to hypothesize a crucial participation of gp78 in liver I/R insult. To confirm the participation of gp78 in acute liver injury, we generated the hepatocyte-specific gp78 knockout (gp78 HKO) mice by crossing gp78-flox mice with Albumin-cre (Alb-cre) transgenic mice. The efficient knockdown of gp78 in livers was confirmed by genotype, qPCR and western blot of the whole liver (Fig. 2A–C). Besides, no significant difference of gp78 expression was observed in other organs, e.g. hearts, kidneys, lungs or spleens of gp78 HKO mice and control mice, indicating that gp78 was specifically knockdown in livers of gp78 HKO mice (Fig. 2B and Fig. S3). After exposure to liver IRI, serum ALT and AST levels were lower in gp78 HKO mice than in WT mice (Fig. 2D). In addition, gp78 HKO mice displayed mitigated liver damage after IR compared with WT mice, as shown by decreased necrotic areas determined by H&E staining (Fig. 2E). Moreover, TUNEL staining and IHC stating for cleaved caspase3 were performed to detect cell death in the ischemic liver lobe sections, and we detected substantially reduced apoptotic cells in gp78 HKO mice after liver IRI (Fig. 2F). These data demonstrated mitigated liver damage in the HKO group after IR. We next explored the relationship between gp78 and inflammation induced by liver IRI. Serum and hepatic mRNA levels of inflammatory cytokines (TNF-α and IL-1β) were inhibited in gp78 HKO mice after reperfusion (Fig. 2G). Similarly, IHC staining for MPO^+^ neutrophils and CD68^+^ macrophages revealed that the numbers of inflammatory cells after I/R were significantly lower in gp78 HKO mice livers than in WT mice livers (Fig. 2H). Moreover, the immunoblot for decreasing cleaved caspase3, cleaved caspase1 and mature-IL-1β also indicated that gp78 deficiency alleviated liver IRI and inflammation in mice undergoing IR (Fig. 2I). In all, these results demonstrated gp78 deficiency in hepatocytes mitigated liver damage and inflammation in mice undergoing IR.

### Hepatocyte-specific gp78 overexpression aggravates liver IRI in mice

**Fig. 3:**
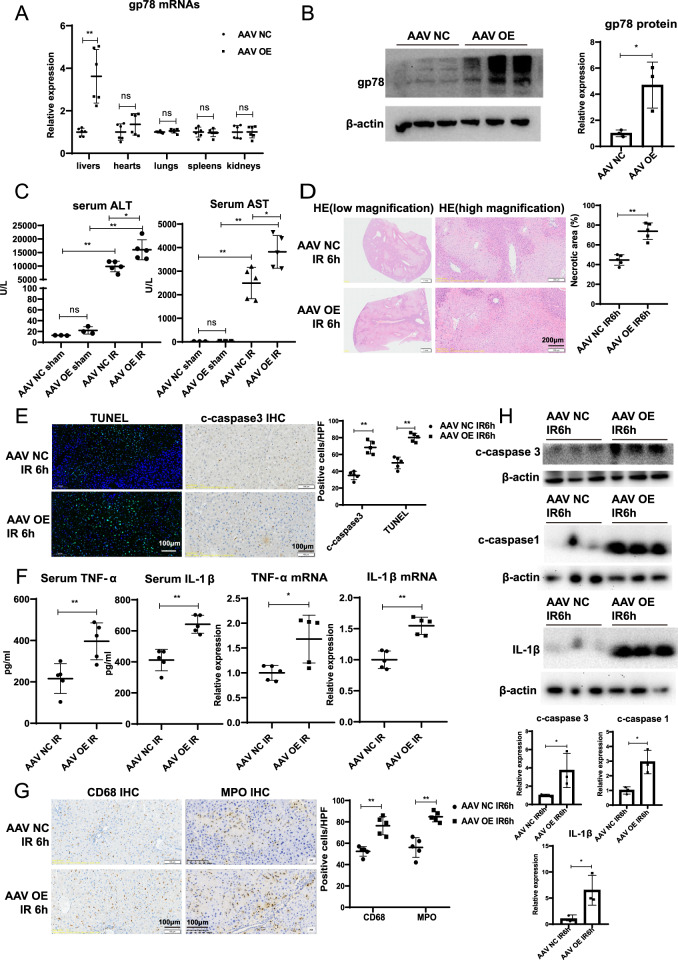
Hepatocyte-specific gp78 overexpression aggravates liver IRI. **A** Gp78 mRNA levels detected by qPCR in the different organs including liver, hearts, kidneys, lungs and spleens from WT and hepatocyte-specific gp78-overexpressed (OE) mice (*n* = 6/group). **B** Gp78 protein levels detected by western blot in the liver from WT and hepatocyte-specific gp78 OE mice (*n* = 3/group). **C** Serum ALT and AST levels in WT and gp78 OE mice at 6 h after I/R injury and sham groups (*n* = 3 for sham groups, *n* = 5 for I/R groups). **D** The representative H&E staining of liver sections from WT and gp78 OE mice in I/R groups at 6 h post reperfusion (*n* = 5/group), scale bar = 200 μm. **E** The representative images of TUNEL staining and IHC staining of cleaved caspase3 in liver sections of WT and gp78 OE mice at 6 h after I/R (*n* = 5/group), scale bar = 100 μm. **F** The levels of Cytokines (TNF-α and IL-1β) in serum and liver of WT and gp78 OE mice at 6 h after I/R (*n* = 5/group). **G** The representative images of immunohistochemistry of CD68 and MPO in liver sections of WT and gp78 OE mice at 6 h after I/R (*n* = 5/group), scale bar = 100 μm. **H** The western blot of cleaved caspase3, cleaved caspase1, mature IL-1β in WT and gp78 OE mice at 6 h after I/R (*n* = 3/group).

To further confirm the role of gp78 in liver IRI, we then generated hepatocyte-specific gp78-overexpressed (gp78 OE) mice by injecting AAV8-TBG-gp78. The efficient overexpression of gp78 in livers was confirmed by qPCR and western blot of the whole livers (Fig. 3A, B). Besides, there was no significant difference of gp78 expression in other organs, e.g. hearts, kidneys, lungs or spleens between gp78 OE mice and control mice (Fig. 3A and Fig. S3). We did not observe significant liver damage in gp78 OE mice under unstressed conditions (Fig. 3C). Notably, compared with WT mice, gp78 OE mice exhibited higher serum ALT and AST levels after liver IR (Fig. 3C), as well as increased necrotic area in histological analysis (Fig. 3D). Additionally, we also detected significantly increased apoptotic cells in gp78 OE mice after reperfusion as determined by TUNEL and IHC staining for cleaved caspase3 (Fig. 3E). These data demonstrated exacerbated liver damage in the gp78 OE mice after IR. Besides, we characterized the effect of hepatic gp78 overexpression on inflammation. Remarkable, gp78 overexpression enhanced the expression of pro-inflammatory cytokines (TNFα and IL-1β) induced by I/R (Fig. 3F). Moreover, IHC staining for MPO^+^ neutrophils and CD68^+^ macrophages revealed that the numbers of inflammatory cells after IR were significantly higher in gp78 OE mice livers than in WT mice livers (Fig. 3G). And the immunoblot for increasing cleaved caspase3, cleaved caspase1 and mature IL-1β in livers also indicated that gp78 promoted liver IRI and hepatic inflammation in vivo (Fig. 3H). Collectively, these results demonstrated gp78 overexpression exacerbated liver damage and inflammation in mice undergoing IR.

### Muti-omics data indicates that gp78 regulates lipid remodeling and ferroptosis

**Fig. 4:**
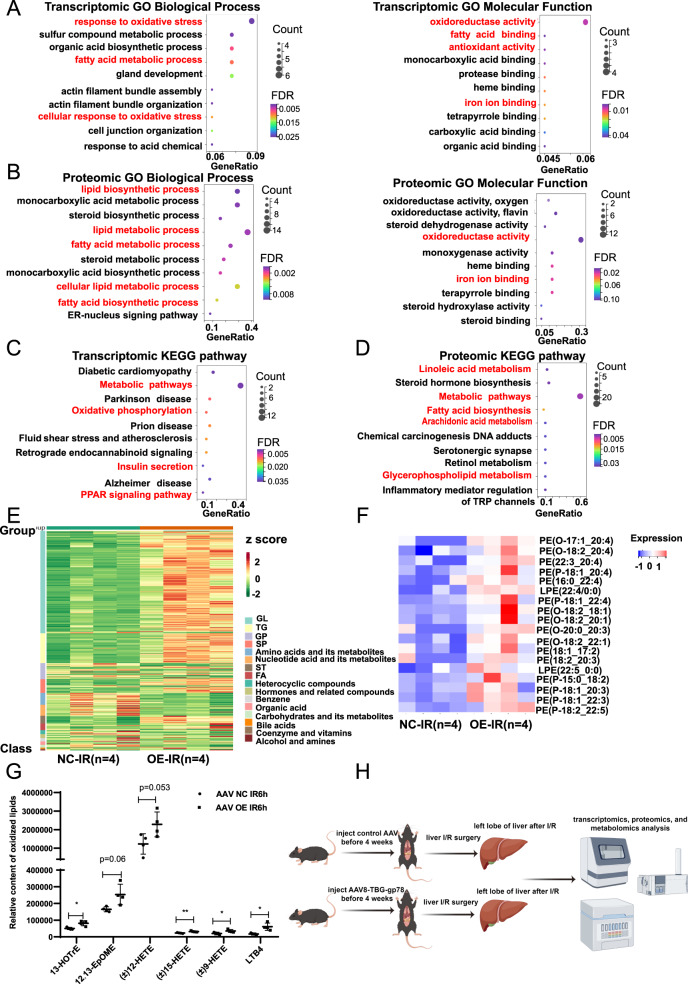
Muti-omics data indicates that gp78 regulates lipid remodeling and ferroptosis. **A** The bubble plot of GO enrichment of transcriptomic results. **B** The bubble plot of GO enrichment of proteomic results. **C** The bubble plot of KEGG enrichment of transcriptomic results. **D** The bubble plot of KEGG enrichment of proteomic results. **E** The heatmaps of dysregulated metabolites in metabolomic analysis. **F** The heatmaps of dysregulated PEs in metabolomic analysis. **G** The levels of oxidized lipids in metabolomic analysis (*n* = 4/group). **H** The experimental design of the muti-omics approach.

To explore the potential mechanism by which gp78 aggravated liver IRI, we performed a multi-omics approach (transcriptomics, proteomics, and metabolomics) to study the characteristics of the liver during IRI (as shown in Fgure 4H). The GO enrichment of transcriptomic and proteomic analysis showed that DEGs were enriched in the pathways associated with ferroptosis, including fatty acid metabolism, response to oxidative stress and iron ion binding (Fig. 4A, B). Besides, the KEGG enrichment of transcriptomic and proteomic analysis showed that lipid metabolic pathways involved in regulation including arachidonic acid (AA) metabolism, linoleic acid (LA) metabolism and glycerophospholipid metabolism were dysregulated between WT mice and gp78 OE mice (Fig. 4C, D), consistent with pathway associated analysis of gp78 (Fig. S4). Moreover, in line with transcriptomic and proteomics analysis, differences in the fatty acid metabolism and ferroptosis pathways were confirmed at the metabolomic level. The untargeted metabolomic revealed significantly different lipid profiles between gp78 OE mice and WT mice. In brief, most glycerolipid (GL), triacylglycerol (TG), sphingolipid (SP), sterol lipids (ST) and fatty acid (FA) were significantly increased in the livers of gp78 OE mice compared with those in WT mice (Fig. 4E). Since ferroptosis is executed by oxidized phospholipids, mainly phosphatidylethanolamines (PEs) that contain PUFA chains (such as AA(C20:4) and AdA(C22:4)), we evaluated the levels of PEs and oxidized lipids in metabolomics. We observed that the levels of PEs with arachidonoyl tail (PE-AA, 20:4) or adrenoyl tail (PE-AdA, 22:4), and AA metabolism products (12-HETE, 15-HETE, LTB4, etc) were increased in gp78 OE mice (Fig. 4F, G). These data suggested that gp78 overexpression regulated cellular lipid remodeling and promoted ferroptosis during liver IRI.

**Fig. 5:**
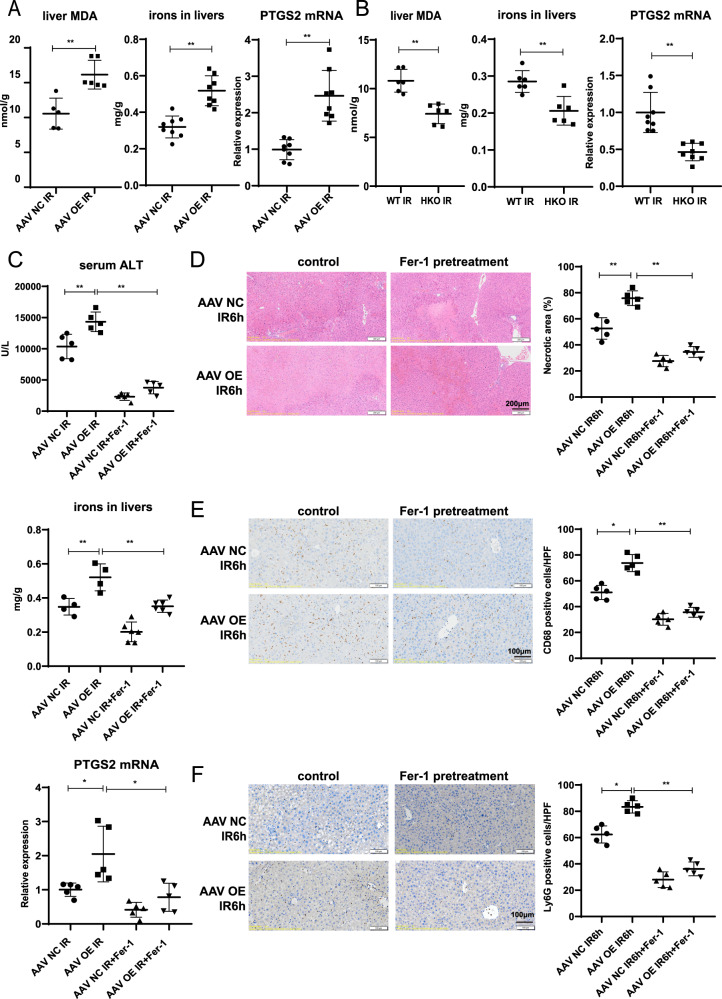
Gp78 promotes liver IRI by regulating ferroptosis. **A** The levels of ferroptosis indicators (MDA, iron and PTGS2 mRNA expression) in livers from WT and gp78 OE mice (*n* = 5–8/group). **B** The levels of ferroptosis indicators (MDA, iron and PTGS2 mRNA expression) in livers from WT and gp78 HKO mice (n = 6–8/group). **C**–**F** Ferrostatin-1 (Fer-1) was i.p. injected 1 h before I/R of gp78 OE and control mice to inhibit ferroptosis, then mice were subjected to I/R surgery. Serum ALT and ferroptosis indicator (iron and PTGS2 mRNA expression) (*n* = 4–6/group) (**C**), Representative H&E staining images (*n* = 5/group), scale bars = 200 μm (**D**), The representative images and the statistical quantification of IHC staining of CD68 and Ly6G (*n* = 5/group), scale bars = 100 μm (**E**, **F**), were evaluated at 6 h after I/R injury in the indicated groups.

To confirm the role of gp78 in ferroptosis, we detected the levels of irons, MDA and expressions of PTGS2 mRNA in the livers. We found increased levels of tissue MDAs, irons and PTGS2 mRNA expressions in gp78 OE mice while those were reduced in gp78 HKO mice compared with those in WT mice (Fig. 5A, B). It indicated that gp78 promoted ferroptosis during liver IR in vivo.

To further confirm whether gp78 promoted liver IRI partly by inducing ferroptosis, we used Ferrostatin-1 (lipid peroxidation inhibitors) before liver IR in WT mice and gp78 OE mice. Ferrostatin-1 pretreatment could improve liver function, reduce the necrotic areas, alleviate ferroptosis and inflammation in gp78 OE mice (Fig. 5C–F). Overall, these results indicated that gp78 promoted liver IRI partly by regulating ferroptosis.

### Gp78 promotes ferroptosis partly by ACSL4

**Fig. 6:**
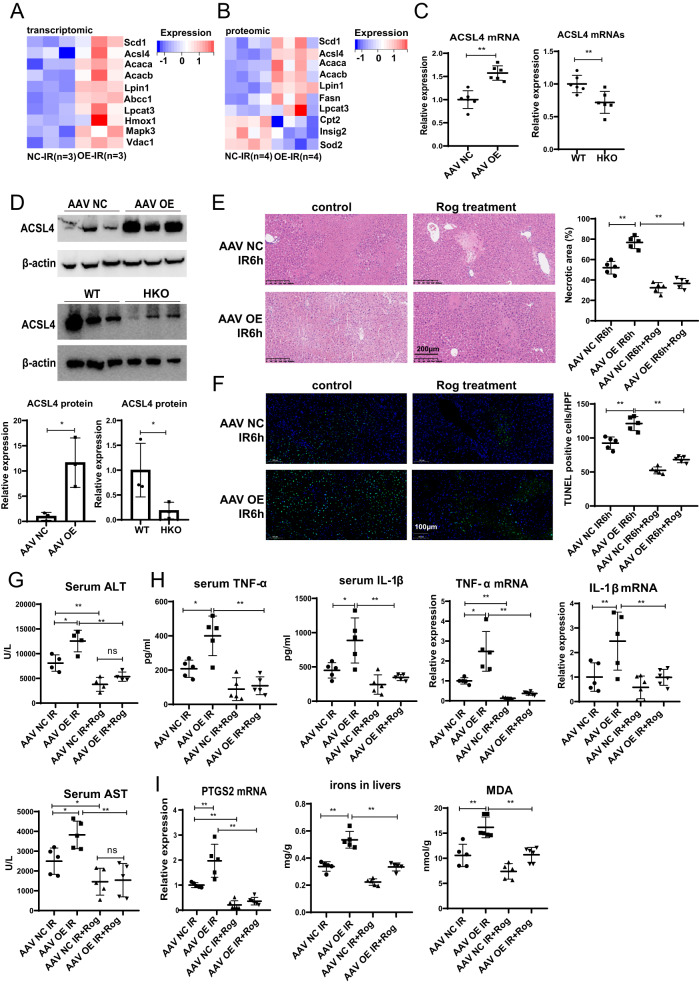
Gp78 regulates ferroptosis partly by regulating ACSL4. **A** The heatmaps of lipid metabolism and ferroptosis genes in the transcriptomic results. **B** The heatmaps of lipid metabolism and ferroptosis genes in the proteomic results. **C**, **D** The mRNA and protein levels of ACSL4 were evaluated in livers from WT and gp78 OE mice or WT and HKO mice (*n* = 6/group for mRNA samples, *n* = 3/group for protein samples). **E**–**I** rosiglitazone (Rog) was i.v. injected 1 h before I/R of gp78 OE and control mice to inhibit ACSL4, then mice were subjected to I/R surgery. The representative H&E staining images, scale bars = 200 μm (**E**), the representative images and the statistical quantification of TUNEL positive cells, scale bars = 100 μm (**F**), serum ALT and AST (**G**), the levels of cytokines (TNF-α and IL-1β) in serum and livers (**H**) and ferroptosis indicators (MDA, iron, PTGS2 mRNA expression) (**I**) were evaluated after I/R injury in the indicated groups. n = 4–6/group.

Further, we explored how gp78 affected ferroptosis by remodeling cellular lipid composition. We investigated the expression of key genes in the changed pathways and found that the expression levels of ACC1, ACSL4, LPCAT3 and other genes related to the fatty acid metabolism and ferroptosis were upregulated in gp78 OE mice compared with WT mice (Fig. 6A, B). ACSL4 has been reported to promote the synthesis of PUFA-containing PEs and induce ferroptosis. We found that ACSL4 expression was elevated in the livers of gp78 OE mice while that was reduced in the livers of gp78 HKO mice (Fig. 6C, D). Methionine and choline deficient (MCD) diet is a common way to induce lipid accumulation in livers. We found that MCD feeding made gp78 OE mice more susceptible to IR-induced liver damage, inflammation and ferroptosis by further disturbing lipid homeostasis. And after IR, the levels of ACSL4 mRNAs and proteins were elevated in gp78 OE mice feeding MCD diets, compared with WT mice feeding MCD diets (Fig. S5). Moreover, a clinically available drug, Rosiglitazone, the inhibitor of ACSL4, was administered to confirm the role of ACSL4 in gp78 OE-induced liver injury. As a result, lower serum ALT and AST, alleviated pathological liver damage and less hepatocyte death were observed after rosiglitazone treatment in gp78 OE mice. Besides, rosiglitazone reduced the levels of pro-inflammatory cytokines (TNF-α and IL-1β) and ferroptosis in gp78 OE mice (Fig. 6E–I). In summary, these results indicated the crucial role of the gp78/ACSL4 axis in ferroptosis and liver IRI.

## Discussion

In this study, we found that the gp78-ACSL4 axis promoted ferroptotic cell death after liver IR, and genetic knockout of gp78 or pharmacologically inhibiting this pathway may provide a new avenue for therapy. Ferroptosis has been reported to play a great role in liver IRI, however, the mechanisms for ferroptosis during IR are not well understood. These findings first demonstrated the importance of gp78 in ferroptosis and liver IRI. Using transcriptomics, proteomics, and metabolomics, we identified gp78 and its downstream partner in fatty acid metabolism, ACSL4, as critical proteins involved in I/R-related ferroptosis, and thus provided a new understanding of disturbed lipid metabolisms driving ferroptosis in liver IRI (Graphical Abstract).

Gp78 is widely expressed, and acts as an E3 ubiquitin ligase at the ER membrane, regulating ER-associated Degradation (ERAD). By regulating the turnover of numerous substrates, gp78 is involved in regulating cell survival, oxidative stress and inflammation process [24–27]. Intriguingly, gp78 is both involved in pro-cell death and anti-cell death pathways. It was reported that gp78 protected the cells from apoptosis by degradation of Bok [25]. In another study, the calcium sensing receptor (CaSR) protected high glucose-induced energy metabolism disorder by blocking the gp78-ubiquitin proteasome pathway [29]. These findings suggest that gp78 can regulate substrates differently in specific settings, similar to the recent report on the dual role of gp78 in inflammation. It was reported that gp78 could bind to NLRP3 directly to restrict inflammasome activation in macrophages [26]. In contrast, gp78 could bind to TAB3 to promote TAK1 activation, promoting the progression of pneumonia [30]. Hence, we should understand the function of gp78 in specific disease backgrounds. Hepatic I/R injury was considered to be sterile inflammation accompanied by multiple cell deaths including ferroptosis. Sensitivity to ferroptosis is determined by lipid remodeling, which provides substrates for lethal lipid peroxidation [31]. For example, phospholipid-modifying enzyme MBOAT1/2 inhibited ferroptosis by increasing monounsaturated fatty acids (MUFAs) specifically [32]. Additionally, lipid flippase solute carrier family 47 member 1 (SLC47A1) regulated lipid remodeling and survival during ferroptosis. Silencing of SLC47A1 increased ferroptosis by favoring ACSL4-SOAT1-mediated production of PUFA-containing cholesterol ester (CE) [33]. Since gp78 could promote lipid synthesis in hepatocytes, we proposed that gp78 might affect ferroptosis by regulating lipid remodeling during liver IR. Herein, we first provided evidence that gp78 deficiency in hepatocytes reduced liver damage and ameliorated inflammation in liver IRI. In muti-omic analysis, gp78 overexpression led to up-regulation of lipogenesis genes, such as ACC1 and ACSL4, causing excess accumulation of lipids, especially PUFA-containing PEs, which could serve as the oxidized substance for ferroptosis during liver IR. Apart from PUFA-containing PEs, the levels of PUFA-containing TGs and PUFA-containing CEs were also elevated in gp78 OE mice, which might also affect ferroptosis. However, not only PUFAs, some MUFAs and saturated fatty acid (SFA) also increase in the metabolomics. The increase of lipids might be caused by SREBP activation upon gp78 degrades insig2, and the levels of diverse lipogenesis genes (SCD1、ACC1、FASN、HMGCR, etc) were elevated in the livers of gp78 OE mice. Lipid accumulation in livers might cause lipotoxicity to hepatocytes, resulting in cell death and inflammation. Thus, PPAR agonist or HMGCR inhibitor (statin) pretreatment might alleviate lipotoxicity and improve the outcomes of LT patients [34].

Our study also strengthens the evidence for the pro-ferroptosis role of ACSL4. ACSL4 belongs to the long-chain fatty acyl-CoA synthetase family (ACSLs), which contains five isoforms identified as ACSL1, 3, 4, 5, and 6 [35]. They convert free long-chain fatty acids into fatty acyl-CoA esters and play an essential role in both anabolic and catabolic pathways. Among these isoforms, ASCL4 is important for the synthesis of PUFA-containg phospholipids, the essential substance of oxidation during ferroptosis [36, 37]. In the study of acute kidney injury (AKI) and intestine IRI, ACSL4 was induced during IRI. Genetic deficiency and chemical inhibitors of ACSL4 could protect the kidney and intestine from IRI [38, 39]. However, its role in liver IRI remains unknown, and a previous study has reported that ACSL4 protein expression was decreased during liver IRI [40]. In our study, ACSL4 expression was increased in gp78 OE mice while that was decreased in gp78 HKO mice, and the ACSL4 inhibitor rosiglitazone could improve liver function and attenuate ferroptosis after liver IR in gp78 OE mice, indicating ACSL4 aggravated ferroptosis and liver IRI.

However, our study also had some limitations. Firstly, we identified that gp78 could affect ACSL4 expression, but we did not clarify how gp78 affects ACSL4 expression. The results of the PPI network of DEGs by String did not indicate the direct interaction between gp78 and ACSL4 (Fig. S6). We proposed that SREBP might be responsible for ACSL4 expression. SREBP could regulate lipid remodeling by transactivating multiple genes involved in lipid metabolism, including ACSL4 (in CHIP-atlas website, chip-atlas.org), and gp78 could activate SREBP by degrading insig1/2. Besides, as a damage-promoting factor, gp78 expression was decreased after LT in our study, it seems contradictory. Maybe loss of gp78 expression was a hepatoprotective response for IR stress. Last but not least, there may be other factors involved in the process of gp78 promoting liver IRI. And further investigation is needed to clarify the direct contribution of lipid remodeling to the hepatic damage caused by gp78.

In conclusion, the current study highlights gp78 as a prominent promoter of hepatic I/R injury. Gp78 could facilitate ACSL4 expression, remodeling PUFA metabolism, leading to ferroptosis and hepatic IRI. Therefore, targeting gp78 or blocking the gp78-ACSL4 axis may represent promising approaches to treat hepatic I/R injury.

### Supplementary information

Supplementary table and supplementary Figure legends

Figure S1

Figure S2

Figure S3

Figure S4

Figure S5

Figure S6

Original Data File

aj-checklist

## Data Availability

The datasets used and/or analyzed during the current study are available from the corresponding author on reasonable request.
