# Supplementary material for: Gp78 deficiency in hepatocytes alleviates hepatic ischemia-reperfusion injury via suppressing ACSL4-mediated ferroptosis
Source: Cell Death Dis. 2023 Dec 8;14(12):810. doi: 10.1038/s41419-023-06294-x (PMC10709349; doi:10.1038/s41419-023-06294-x)
Supplement: Supplementary file 1 — Supplementary table and supplementary Figure legends [file 41419_2023_6294_MOESM1_ESM.doc]

Table S1. The information of clinical samples

|  | Liver transplantation |
| --- | --- |
| Age (years) | 48.1±10.9 |
| Gender(M/F) | 29/6 |
| Time of biopsy | 2022.3.10-2022.10.30 |
| Medical history | All donors were not diagnosed with hepatitis and steatosis |
| Donation Status | Donation after brain death |

Table S2. The primers for genotyping and real-time PCR analysis:

| Gene | Primer (5′-3′) |
| --- | --- |
| Gp78 flox Forward | CCTCCTGTCCTGAGAGTATTAGTGT |
| Gp78 flox Reverse | AACAAAGGAATCAAACCCCAAA |
| Alb cre Forward | TGGATGCCACCTCTGATGAAGTC |
| Alb cre Reverse | TCCTGGCATCTGTCAGAGTTCTCC |
| β-actin Forward | GTGACGTTGACATCCGTAAAGA |
| β-actin Reverse | GCCGGACTCATCGTACTCC |
| Tnfa Forward | CAGGCGGTGCCTATGTCTC |
| Tnfa Reverse | CGATCACCCCGAAGTTCAGTAG |
| Il1b Forward | GAAATGCCACCTTTTGACAGTG |
| Il1b Reverse | TGGATGCTCTCATCAGGACAG |
| Il6 Forward | CTGCAAGAGACTTCCATCCAG |
| Il6 Reverse | AGTGGTATAGACAGGTCTGTTGG |
| Mcp-1 Forward | TAAAAACCTGGATCGGAACCAAA |
| Mcp-1 Reverse | GCATTAGCTTCAGATTTACGGGT |
| Ptgs2 Forward | TTCCAATCCATGTCAAAACCGT |
| Ptgs2 Reverse | AGTCCGGGTACAGTCACACTT |
| Acsl4 Forward | CCTGAGGGGCTTGAAATTCAC |
| Acsl4 Reverse | GTTGGTCTACTTGGAGGAACG |

Table S3: Antibodies for western blot and IHC:

| Antibodies | Source | Cat NO |
| --- | --- | --- |
| β-actin （for western blot） | Abclonal | AC038 |
| Cleaved-caspase3（for western blot） | CST | 9664 |
| Cleaved-IL-1β（for western blot） | CST | 63124 |
| ACSL4（for western blot） | Abclonal | A20414 |
| Gp78（for western blot and IHC） | proteintech | 16675-1-AP |
| Cleaved-caspase3（for IHC） | Servicebio | GB11532 |
| MPO（for IHC） | Servicebio | GB11224 |
| CD68（for IHC） | Servicebio | GB113109 |
| Ly6g（for IHC） | Servicebio | GB11229 |

**Supplenmentary Figure legends**

**Figure S1.** IHC scores of every liver biopsy of donors before transplantation.

**Figure S2.** The expression of gp78 in single cells of livers according to The Human Protein Atlas.

**Figure S3.** The protein expressions of gp78 in hearts，kidneys， lungs and spleens from AAV-gp78 overexpression mice, gp78 hepatocyte specific knockout mice and control mice (n=3).

**Figure S4.** Pathway associated analysis of gp78 in TCGA database on the websites of assistant for clinical bioinformatics (www.aclbi.com).

**Figure S5.** Methionine-choline deficiency (MCD) diets feeding makes gp78 OE mice more susceptible to IR induced liver damage, inflammation and ferroptosis. Gp78 OE mice and WT mice were fed MCD diets for 4 weeks to induce lipid accumulation in livers, then mice were subjected to I/R surgery（n=6/group）. Serum ALT and AST (A), the mRNA expression of inflammatory cytokines (TNF-α、IL-1β、IL-6、MCP-1) and ferroptosis genes (ACSL4 and PTGS2) (B), the irons in livers (C), the protein expression of ACSL4 (D) were evaluated after I/R injury in the indicated groups （n=3/group).

**Figure S6.** The PPI network of DEGs of the proteomic results by String.
