# Supplementary figures and images for: Gp78 deficiency in hepatocytes alleviates hepatic ischemia-reperfusion injury via suppressing ACSL4-mediated ferroptosis
Source: Cell Death Dis. 2023 Dec 8;14(12):810. doi: 10.1038/s41419-023-06294-x (PMC10709349; doi:10.1038/s41419-023-06294-x)

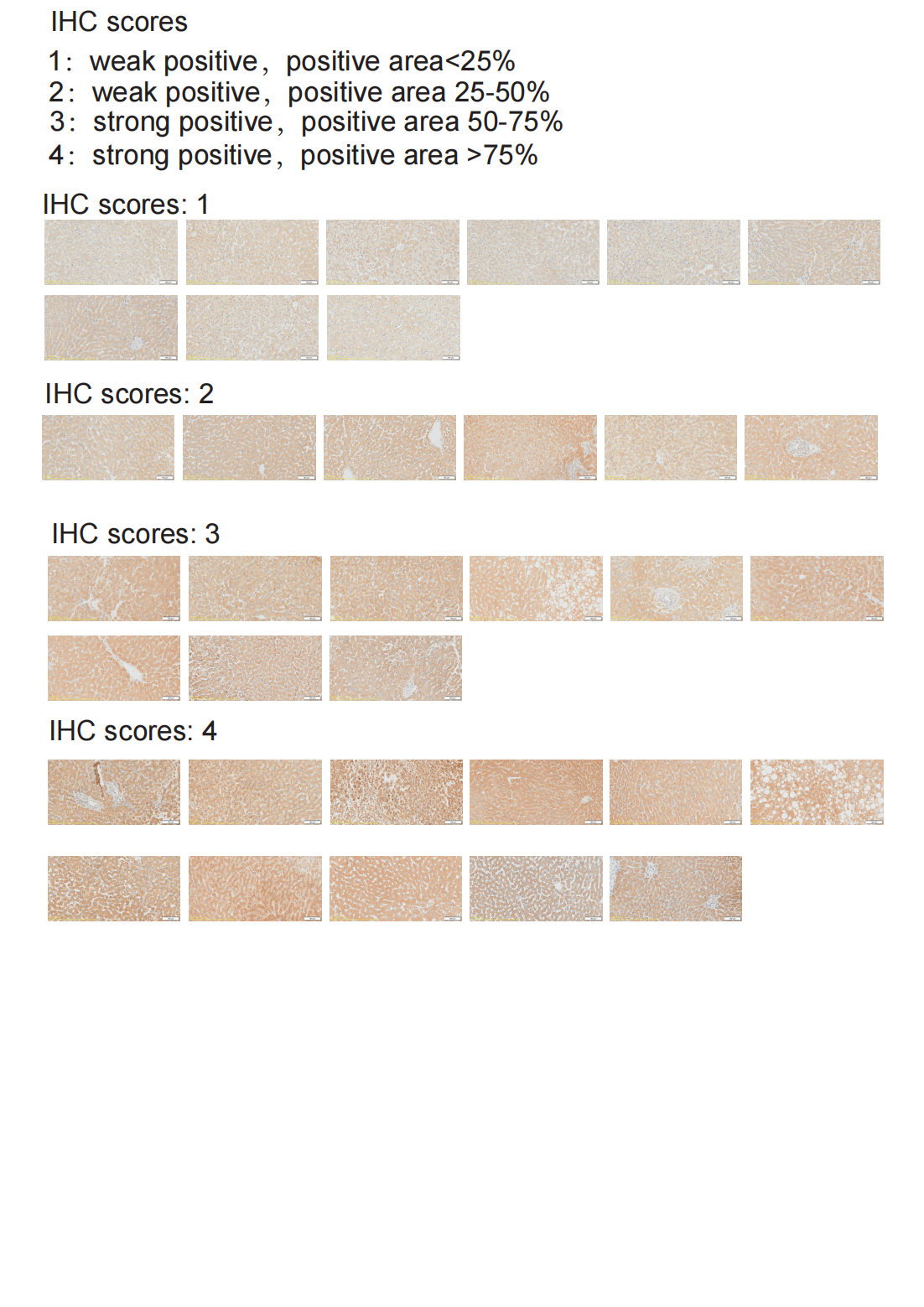

Supplement: Supplementary file 2 — Figure S1 [file 41419_2023_6294_MOESM2_ESM.tif]

Max-norm Z-score  
log(nTPM) z-score

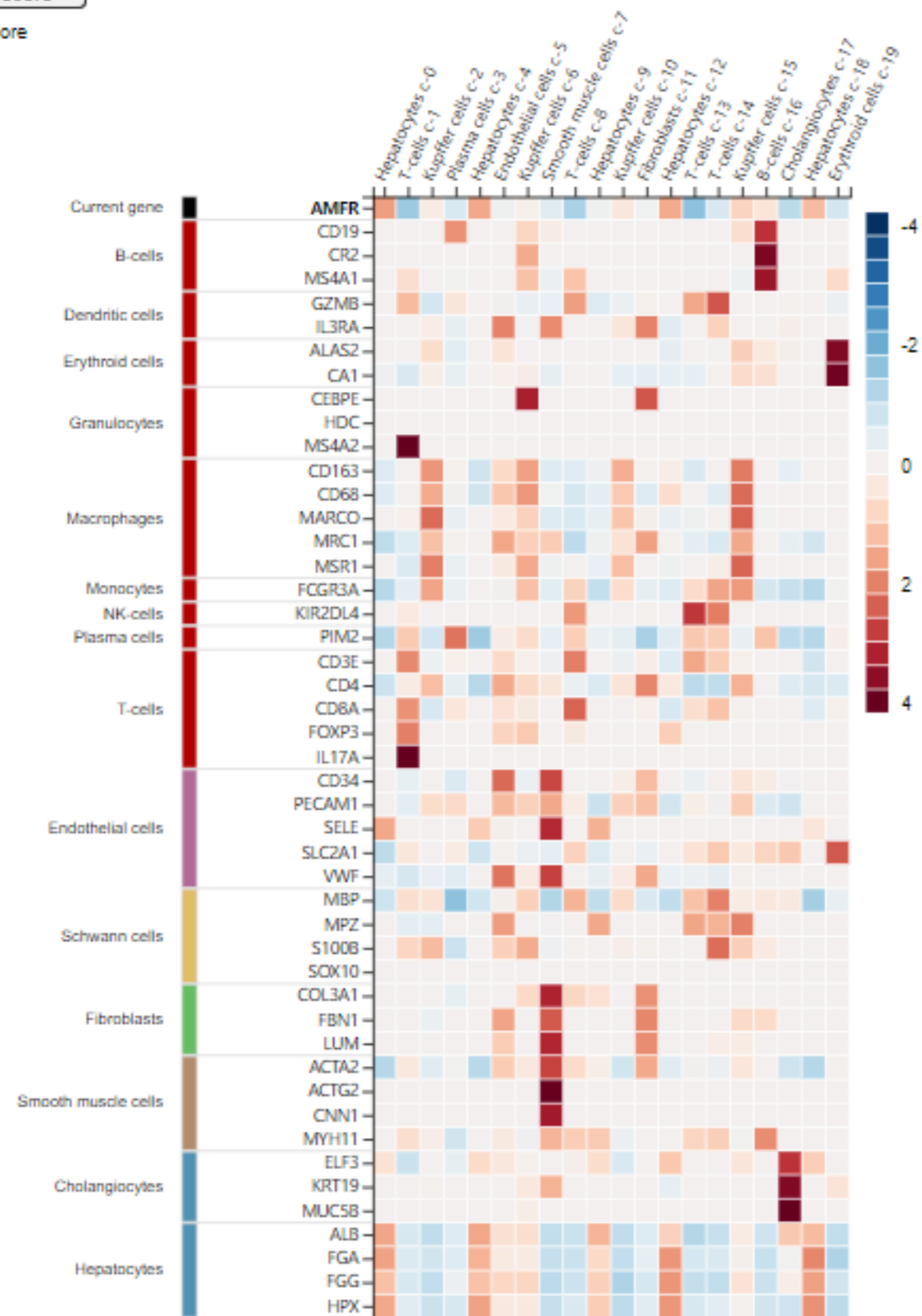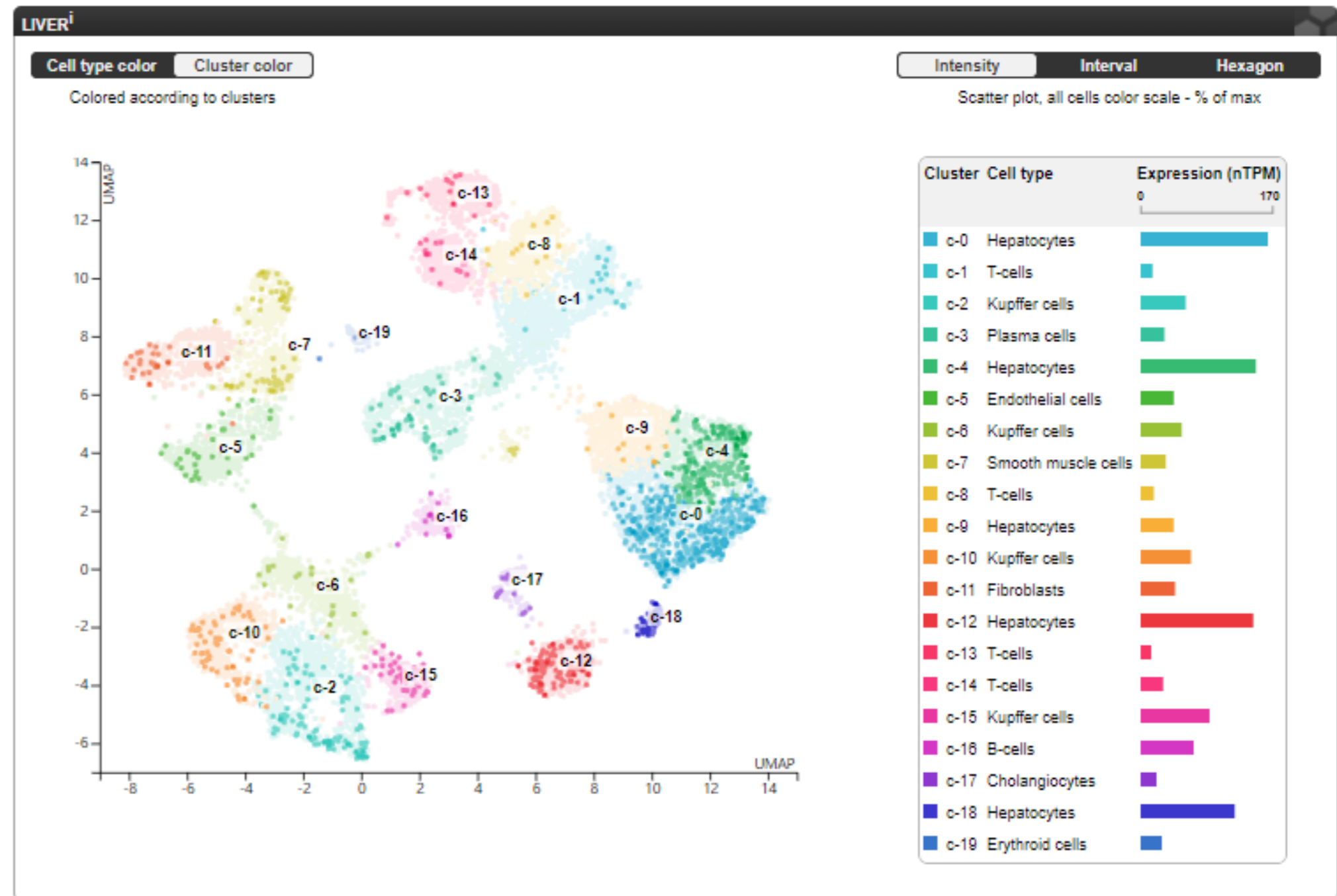

Supplement: Supplementary file 3 — Figure S2 [file 41419_2023_6294_MOESM3_ESM.pdf]

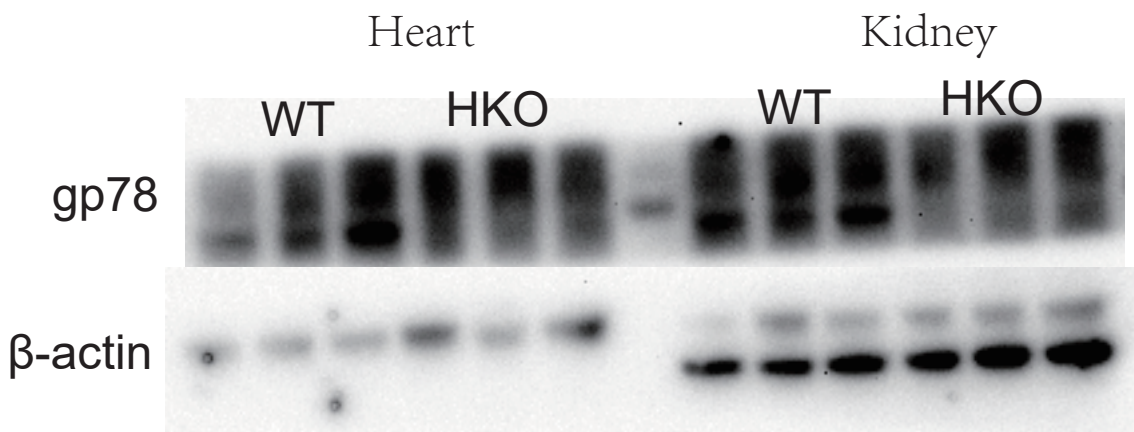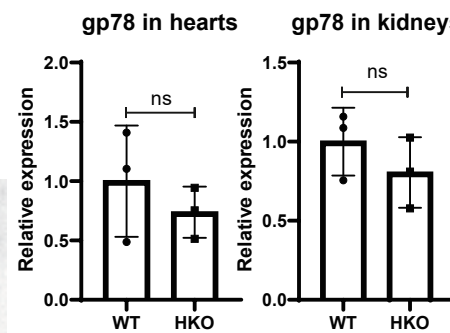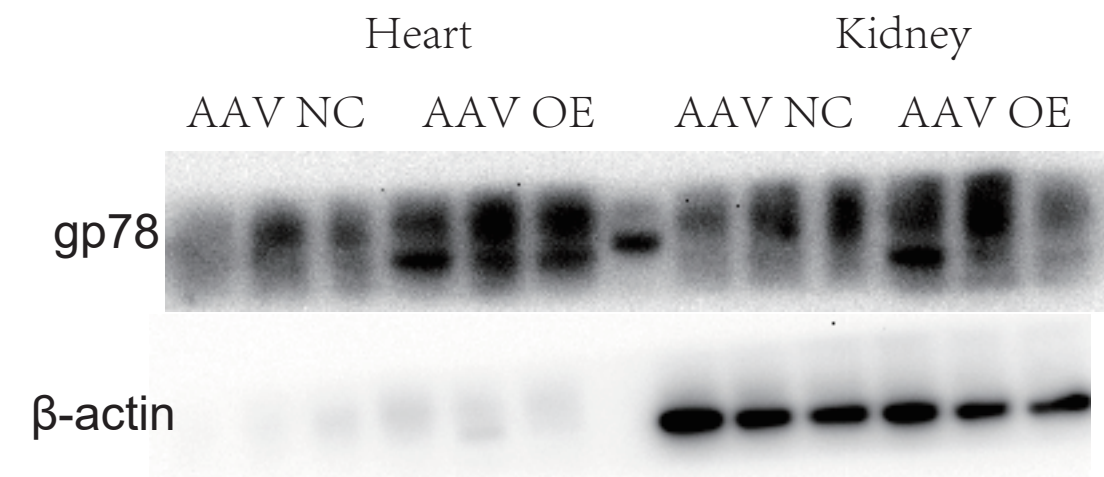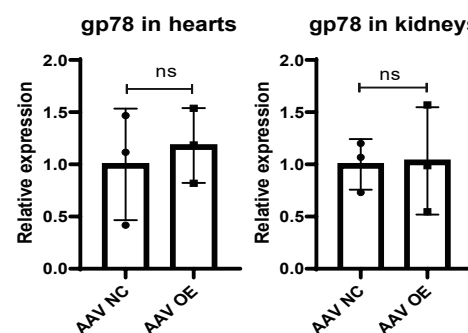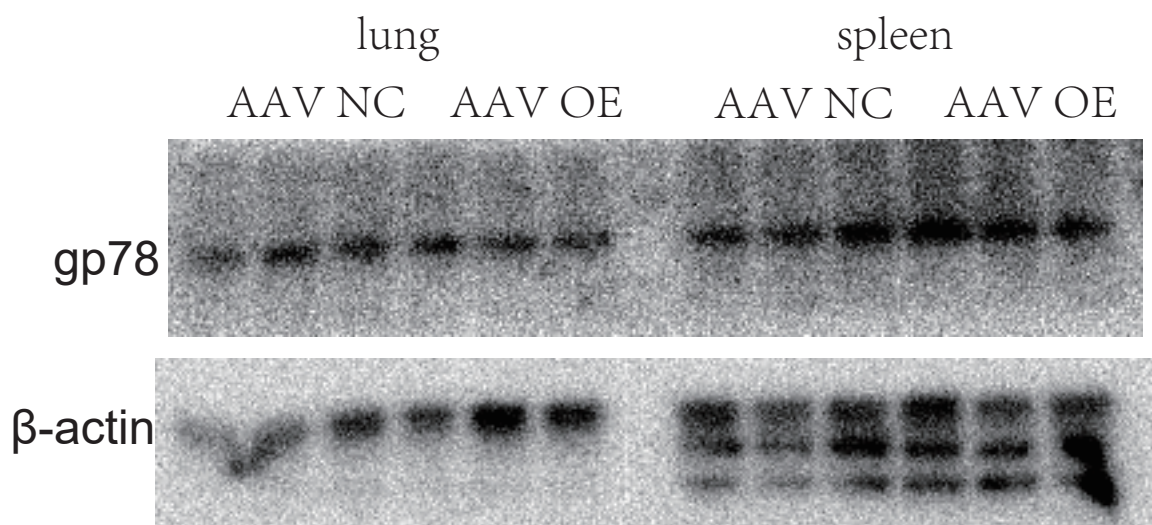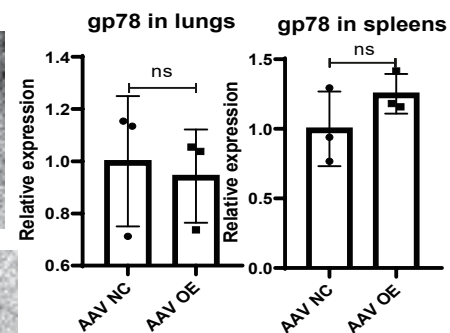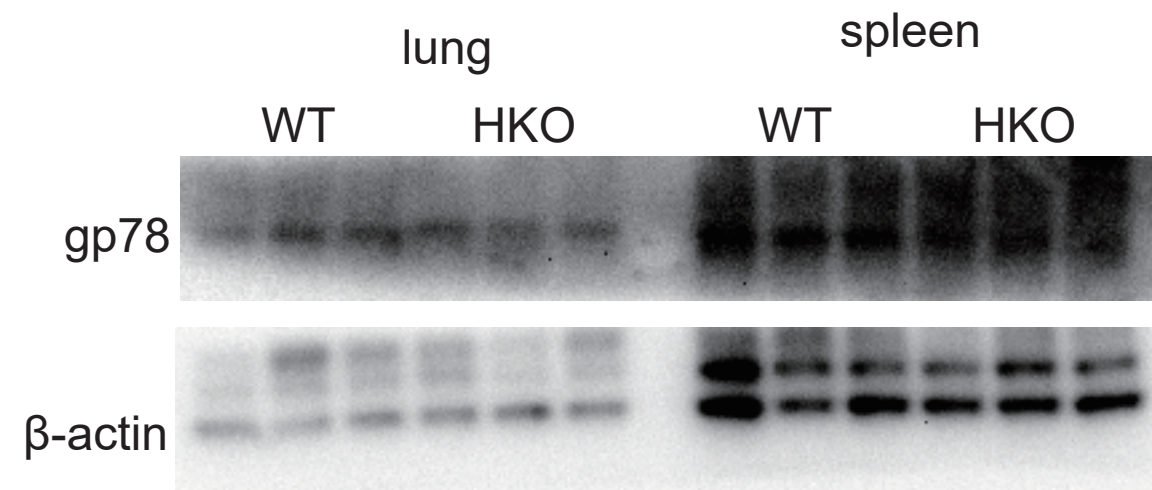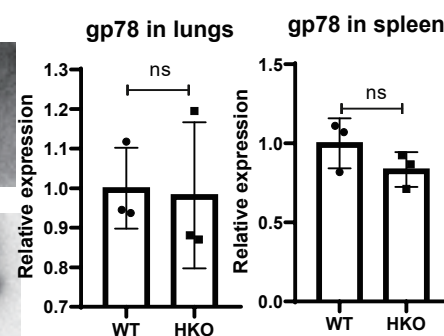

Supplement: Supplementary file 4 — Figure S3 [file 41419_2023_6294_MOESM4_ESM.pdf]

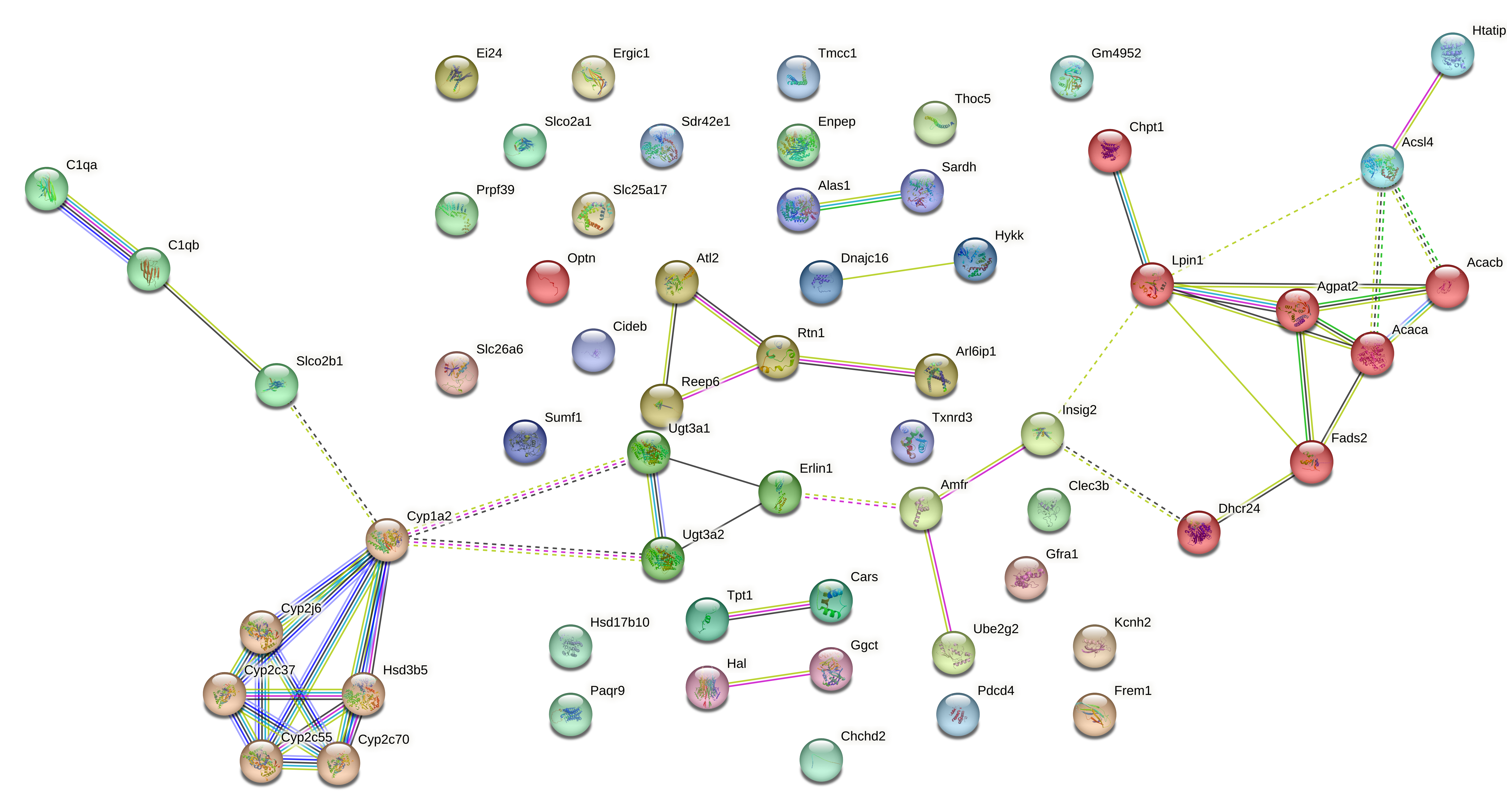

Supplement: Supplementary file 7 — Figure S6 [file 41419_2023_6294_MOESM7_ESM.png]
