# Supplementary material for: Gp78 deficiency in hepatocytes alleviates hepatic ischemia-reperfusion injury via suppressing ACSL4-mediated ferroptosis
Source: Cell Death Dis. 2023 Dec 8;14(12):810. doi: 10.1038/s41419-023-06294-x (PMC10709349; doi:10.1038/s41419-023-06294-x)
Supplement: Supplementary file 5 — Figure S4 [file 41419_2023_6294_MOESM5_ESM.pdf]

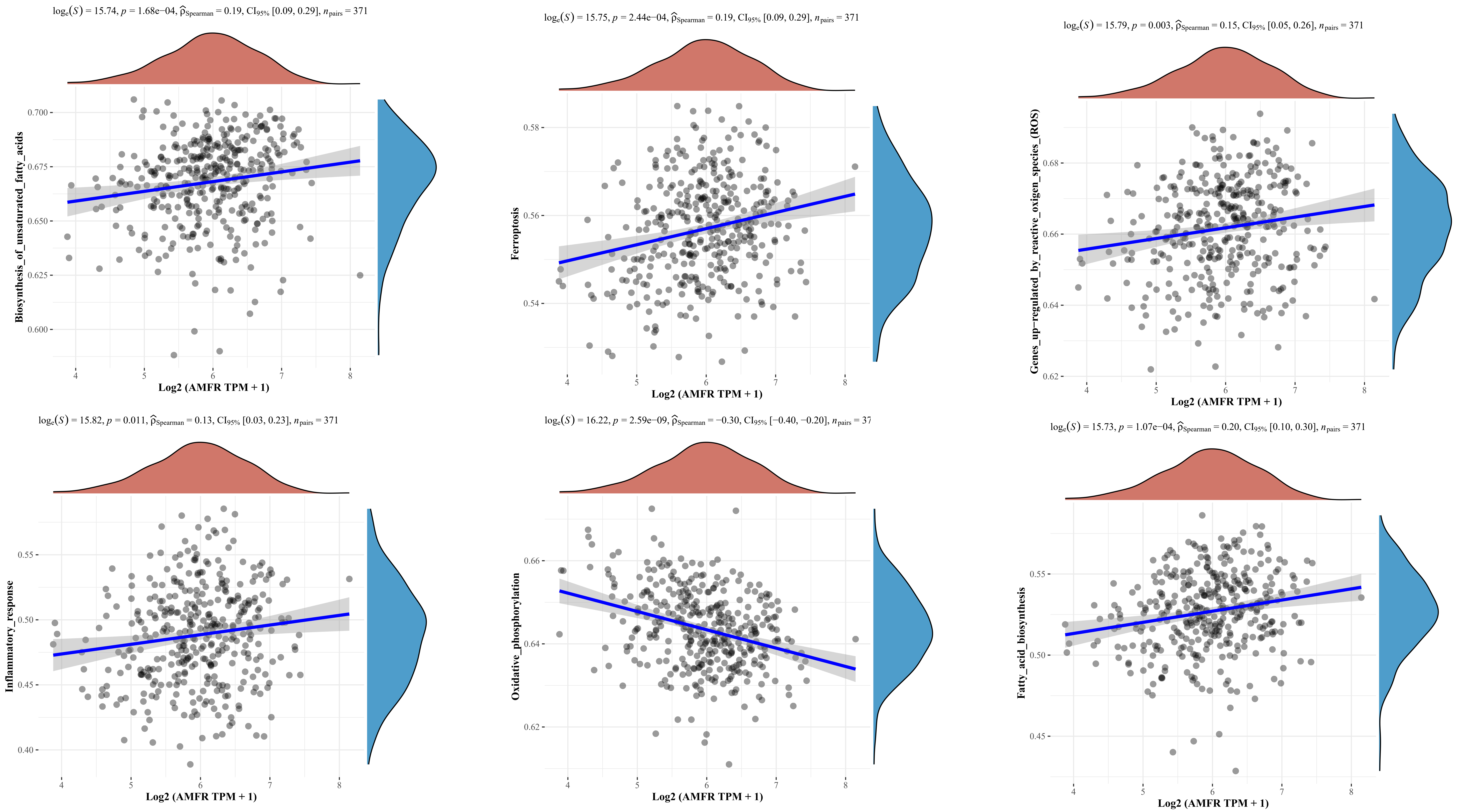

$\log_e(S) = 15.74, p = 1.68e{-04}, \widehat{\rho}_{Spearman} = 0.19, CI_{95\%} [0.09, 0.29], n_{pairs} = 371$
Biosynthesis_of_unsaturated_fatty_acids
Log2 (AMFR TPM + 1)
$\log_e(S) = 15.75, p = 2.44e{-04}, \widehat{\rho}_{Spearman} = 0.19, CI_{95\%} [0.09, 0.29], n_{pairs} = 371$
Ferroptosis
Log2 (AMFR TPM + 1)
$\log_e(S) = 15.79, p = 0.003, \widehat{\rho}_{Spearman} = 0.15, CI_{95\%} [0.05, 0.26], n_{pairs} = 371$
Genes_up−regulated_by_reactive_oxigen_species_(ROS)
Log2 (AMFR TPM + 1)
$\log_e(S) = 15.82, p = 0.011, \widehat{\rho}_{Spearman} = 0.13, CI_{95\%} [0.03, 0.23], n_{pairs} = 371$
Inflammatory_response
Log2 (AMFR TPM + 1)
$\log_e(S) = 16.22, p = 2.59e{-09}, \widehat{\rho}_{Spearman} = -0.30, CI_{95\%} [-0.40, -0.20], n_{pairs} = 37$
Oxidative_phosphorylation
Log2 (AMFR TPM + 1)
$\log_e(S) = 15.73, p = 1.07e{-04}, \widehat{\rho}_{Spearman} = 0.20, CI_{95\%} [0.10, 0.30], n_{pairs} = 371$
Fatty_acid_biosynthesis
Log2 (AMFR TPM + 1)
