# Supplementary material for: Gp78 deficiency in hepatocytes alleviates hepatic ischemia-reperfusion injury via suppressing ACSL4-mediated ferroptosis
Source: Cell Death Dis. 2023 Dec 8;14(12):810. doi: 10.1038/s41419-023-06294-x (PMC10709349; doi:10.1038/s41419-023-06294-x)
Supplement: Supplementary file 6 — Figure S5 [file 41419_2023_6294_MOESM6_ESM.pdf]

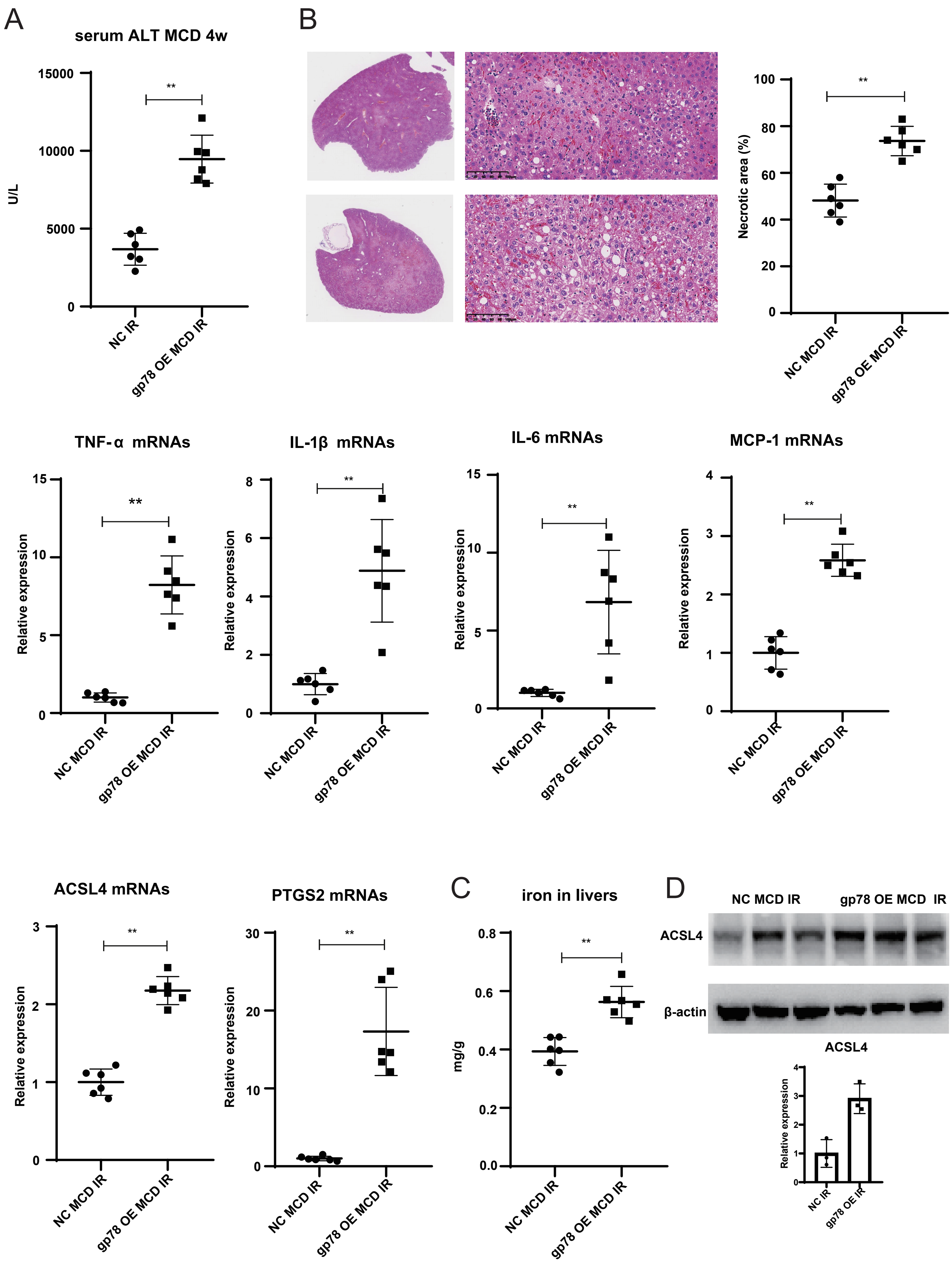

A
serum ALT MCD 4w
U/L
15000
10000
5000
0
**
NC IR
gp78 OE MCD IR
B
Necrotic area (%)
100
80
60
40
20
0
**
NC MCD IR
gp78 OE MCD IR
TNF-α mRNAs
IL-1β mRNAs
IL-6 mRNAs
MCP-1 mRNAs
Relative expression
**
NC MCD IR
gp78 OE MCD IR
ACSL4 mRNAs
PTGS2 mRNAs
C
iron in livers
mg/g
0.8
0.6
0.4
0.2
0.0
D
NC MCD IR
gp78 OE MCD IR
ACSL4
β-actin
ACSL4
Relative expression
NC IR
gp78 OE IR
