# Supplementary material for: Gp78 deficiency in hepatocytes alleviates hepatic ischemia-reperfusion injury via suppressing ACSL4-mediated ferroptosis
Source: Cell Death Dis. 2023 Dec 8;14(12):810. doi: 10.1038/s41419-023-06294-x (PMC10709349; doi:10.1038/s41419-023-06294-x)
Supplement: Supplementary file 8 — Original Data File [file 41419_2023_6294_MOESM8_ESM.pptx]

## Slide 1
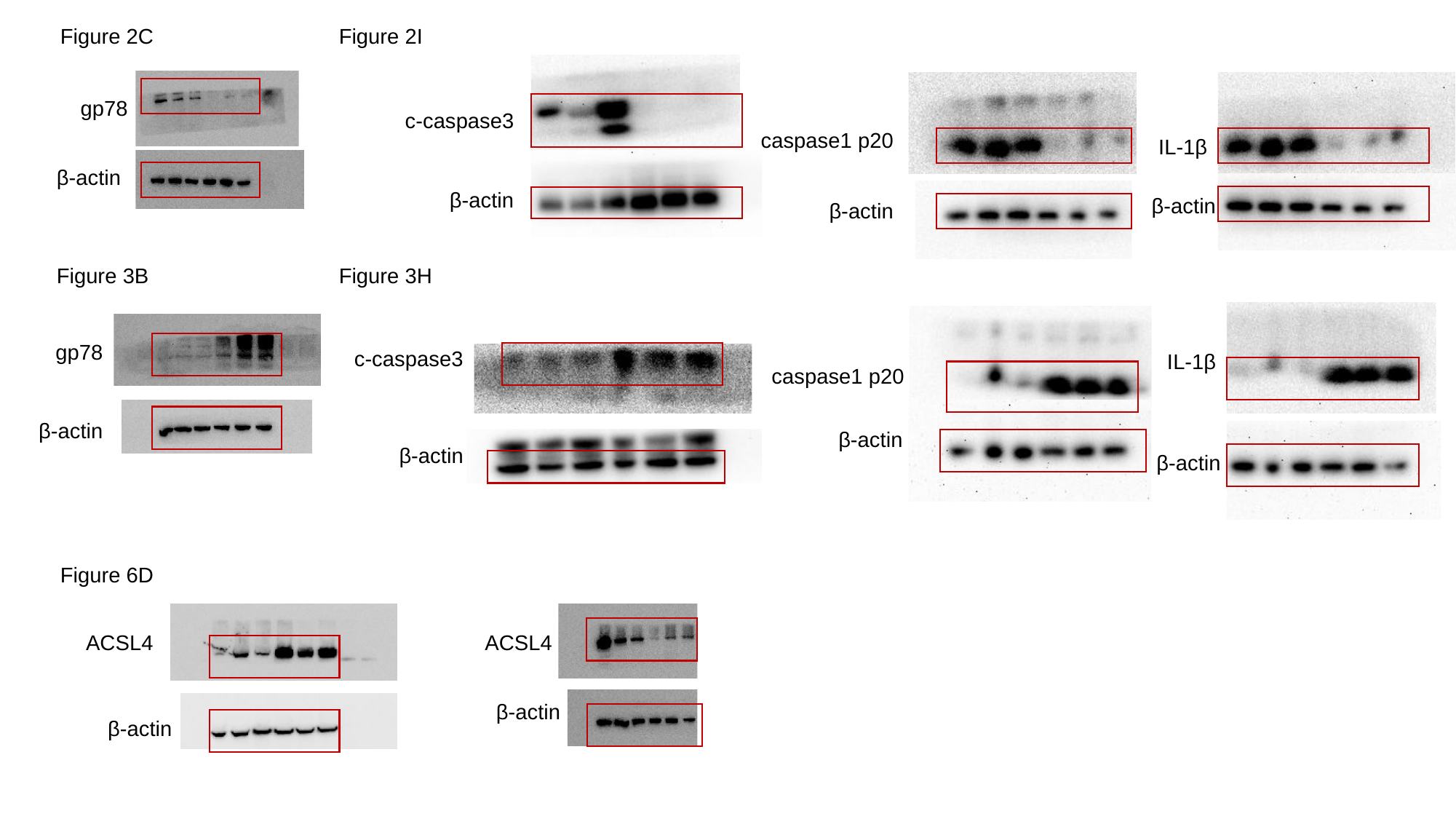

Figure 2C
Figure 2I
β-actin
gp78
c-caspase3
caspase1 p20
 IL-1β
β-actin
β-actin
β-actin
Figure 3B
Figure 3H
IL-1β
β-actin
caspase1 p20
β-actin
gp78
β-actin
c-caspase3
β-actin
Figure 6D
ACSL4
β-actin
ACSL4
β-actin

## Slide 2
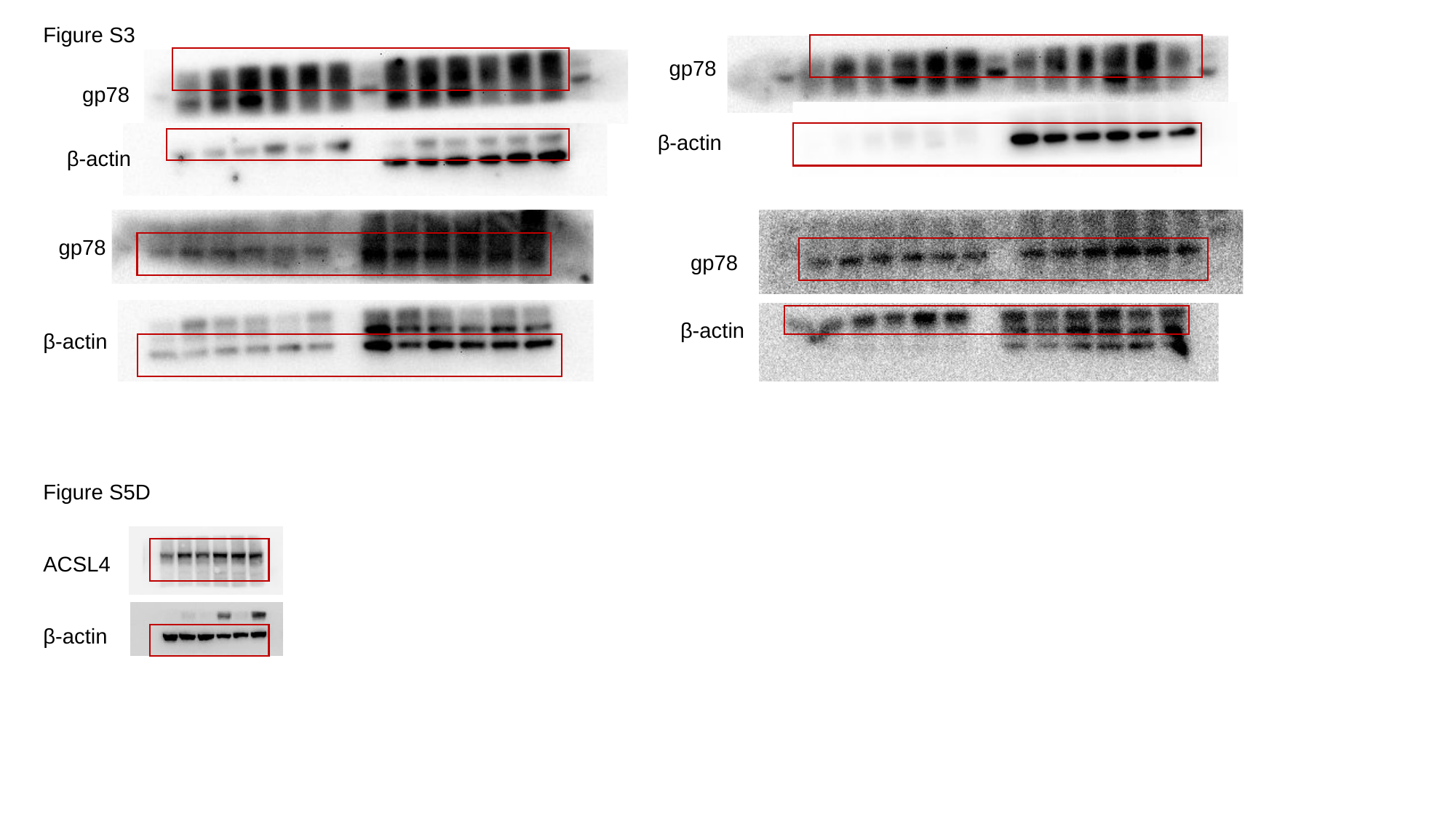

Figure S3
gp78
gp78
β-actin
β-actin
gp78
gp78
β-actin
β-actin
Figure S5D
ACSL4
β-actin
